# The Effect of Intravenous Lidocaine on Duodenal Peristalsis and Sedation Quality During Endoscopic Retrograde Cholangiopancreatography: A Randomized Controlled Trial

**DOI:** 10.5152/tjg.2026.26266

**Published:** 2026-07-08

**Authors:** Mehmet Şahap, Önder Aydemir, Kerem Kenarlı, Bülent Ödemiş, Abdulkadir But

**Affiliations:** 1 Department of Anesthesiology and Reanimation, Ankara Bilkent City Hospital, Ankara Yıldırım Beyazıt University Faculty of Medicine, Ankara, Türkiye; 2 Provincial Health Directorate, Konya, Türkiye; 3 Department of Gastroenterology, Ankara Bilkent City Hospital, Ankara, Türkiye

**Keywords:** Cholangiopancreatography, deep sedation, endoscopic retrograde, ERCP, lidocaine, propofol, sedation

## Abstract

**Background/Aims:**

The current study aimed to evaluate the effects of intravenous (IV) lidocaine on sedative requirements, duodenal peristalsis, and procedural outcomes in patients undergoing endoscopic retrograde cholangiopancreatography (ERCP) under deep sedation.

**Materials and Methods:**

In this prospective, randomized, double-blind, placebo-controlled study, 120 patients with ASA I–III who were scheduled for elective ERCP were enrolled. Participants were randomized to 2 groups: the lidocaine group (n = 60) received a 1 mg/kg bolus followed by a 2 mg/kg/h infusion, whereas the control group (n = 60) received an equivalent volume of 0.9% saline. Sedation was maintained with propofol and titrated to a Bispectral Index of 60-80. Duodenal peristalsis was assessed using a predefined semiquantitative peristalsis scoring system.

**Results:**

Demographic characteristics were similar between groups. Total propofol consumption was significantly lower in the lidocaine group (*P* < .001). Recovery and discharge times were significantly shorter in the lidocaine group than in the control group (*P* < .001 for both). Clinically adequate inhibition of duodenal peristalsis was observed more frequently in the lidocaine group (90.0% vs. 75.0%; *P* = .031), and the requirement for rescue antispasmodics was lower (*P* = .018). Complication rates, cannulation time, primary success rate, and endoscopist satisfaction were similar between groups.

**Conclusion:**

IV lidocaine may serve as a supportive anesthetic-sparing adjunct during ERCP, reducing propofol requirements and facilitating shorter recovery times. Although lidocaine was associated with greater inhibition of duodenal peristalsis and reduced use of rescue antispasmodic, major procedural outcomes were comparable between groups; therefore, these findings should be interpreted cautiously.

**Main Points**

- Intravenous (IV) lidocaine was associated with more frrequent achievement of clinically adequate inhibition of duodenal peristalsis during endoscopic retrograde cholangiopancreatography (ERCP).
- Systemic lidocaine administration reduced total propofol requirements during deep sedation.
- Lidocaine use was associated with shorter recovery and discharge times.
- IV lidocaine reduced the need for rescue antispasmodic agents during ERCP.

## Introduction

Endoscopic retrograde cholangiopancreatography (ERCP) is a well-established procedure for the diagnosis and treatment of biliary and pancreatic duct diseases; however, it remains one of the most technically challenging endoscopic procedures.^1^ The success of ERCP depends on accurate and timely cannulation of the major duodenal papilla, which is influenced by patient compliance under deep sedation and effective control of duodenal peristalsis.^2^

Duodenal peristalsis and spasms occurring during ERCP can impair endoscopic visualization, prolong cannulation time, increase the risk of cannulation failure, and indirectly increase radiation exposure for both patients and medical staff.^3^^,^^4^

In clinical practice, antimuscarinic agents such as hyoscine-N-butylbromide and glucagon are commonly used to suppress duodenal peristalsis. However, their use may be limited by adverse effects, including tachycardia, exacerbation of glaucoma, urinary retention, and xerostomia, particularly in older adults and patients with limited cardiac reserve.^5^ In addition, glucagon should be used with caution because it may cause hyperglycemia and electrolyte disturbances, particularly in patients with diabetes.^6^ These limitations highlight the need for alternative pharmacological approaches that provide effective duodenal relaxation while minimizing anticholinergic adverse effects.

Intravenous (IV) lidocaine is widely used as part of multimodal analgesia protocols because of its anti-inflammatory, analgesic, and antihyperalgesic properties.^7^^-^^9^ In addition to blocking sodium channels, lidocaine has been reported to modulate visceral reflex activity, influence gastrointestinal peristalsis, and reduce visceral reflexes such as gagging, coughing, and hiccups, suggesting a potential spasmolytic role during endoscopic procedures.^10^

Studies investigating the inhibition of duodenal peristalsis during ERCP are limited, and existing data have primarily focused on topical lidocaine. However, the technical limitations and short duration of action associated with topical administration highlight the potential advantages of systemic administration. To the best of the authors’ knowledge, prospective studies evaluating the effects of IV lidocaine on duodenal peristalsis and sedation quality during ERCP are limited.

The current study prospectively evaluated the effects of IV lidocaine on duodenal peristalsis, sedative requirements, hemodynamic parameters, and selected procedural outcomes during ERCP performed under deep sedation.

## Materials and Methods

### Study Design and Ethical Approval

This prospective, randomized, double-blind, placebo-controlled clinical trial was conducted through a collaboration between the Department of Anesthesiology and Reanimation and the Department of Gastroenterology at a tertiary care hospital. The study protocol was approved by the Ankara Bilkent City Hospital Clinical Research Ethics Committee (approval no: E2-26-14071, date: January 21, 2026) and registered at ClinicalTrials.gov (NCT07333859). The study was conducted and reported in accordance with the Consolidated Standards of Reporting Trials guidelines. Written informed consent was obtained from all participants in accordance with the principles of the Declaration of Helsinki.

### Patient Population

Patients aged 18 to 85 years with an American Society of Anesthesiologists (ASA) physical status of I-III who were scheduled for elective ERCP were included. Exclusion criteria included allergy to amide-type local anesthetics, severe hepatic impairment (Child–Pugh class C), advanced renal failure, high-degree atrioventricular block, pregnancy, active neurological disease, and refusal to participate.

### Randomization and Blinding

Patients were randomized to 2 groups: the lidocaine group (group L; n = 60) and the control group (group C; n = 60) using a computer-generated randomization sequence. Study medications (lidocaine or 0.9% NaCl) were prepared in identical volumes and appearances by an independent anesthesia technician who was not involved in the study. Anesthesia management was performed by an anesthesiologist blinded to group allocation. The primary outcome (duodenal peristalsis score) was assessed by a blinded endoscopist, and statistical analyses were performed by an independent researcher blinded to group assignment.

### Anesthetic Management and Protocol

Standard monitoring (electrocardiography (ECG), noninvasive blood pressure, and SpO_2_) and Bispectral Index (BIS) monitoring were applied to all patients. Sedation was induced with 0.5-1 mg/kg propofol. Maintenance was achieved with a propofol infusion of 40-60 µg/kg/min, titrated to maintain a BIS value of 60-80.

- Group L (lidocaine group): Patients received an IV bolus of lidocaine (1 mg/kg) 2 minutes before induction, followed by a continuous infusion of 2 mg/kg/h throughout the procedure.
- Group C (control group): Patients received an equivalent volume of 0.9% saline administered at the same rate throughout the procedure.

When inadequate sedation or patient movement occurred during the procedure, supplemental propofol boluses were administered at the discretion of the attending anesthesiologist to maintain the target BIS range and optimal procedural conditions. All supplemental propofol doses were included in the analysis of total propofol consumption. No adjunctive analgesic agents were used as part of the study protocol.

After visualization of the papilla, duodenal peristalsis was assessed. If significant peristalsis was observed, pharmacological intervention was initiated. Hyoscine-N-butylbromide was used as the first-line antispasmodic agent; glucagon was used when contraindications to hyoscine-N-butylbromide existed or as adjunctive therapy when a single agent was insufficient.

### Variables and Data Collection

#### Assessment of Duodenal Peristalsis:

Duodenal peristalsis was assessed by the endoscopist using a 5-point semiquantitative scale adapted from previously published endoscopic peristalsis assessment studies. Scores ranged from 1, indicating severe peristalsis with continuous contractions that prevented cannulation, to 5, indicating complete duodenal quiescence. Scores of 4 or 5 were considered indicative of clinically adequate inhibition of duodenal peristalsis, representing minimal or absent peristaltic activity unlikely to interfere with endoscopic cannulation, whereas scores of 1 to 3 were classified as inadequate inhibition. Although similar scales have been used in previous ERCP and colonoscopy studies, this scoring system has not been formally validated. ^11^

#### Technical and Clinical Parameters:

Cannulation time (seconds), presence of a native papilla, primary cannulation success rate, cannulation difficulty, and the dose of rescue antispasmodics were recorded. 

#### Hemodynamic and Sedation Parameters:

Heart rate, mean arterial pressure (MAP), SpO_2_, BIS, and Ramsay Sedation Scale scores were recorded at baseline, after induction, at 5, 10, 15, 20, and 30 minutes, and at the end of the procedure.

#### Safety and Recovery:

The occurrence of visceral reflexes (gagging, coughing, and hiccups), involuntary movements, and adverse events (hypotension, bradycardia, hypoxemia, nausea, and vomiting) was documented. Endoscopist satisfaction (0-10 Numeric Rating Scale (NRS)), recovery time (Modified Aldrete Score ≥9), and the presence of sore throat at 2 hours after the procedure were evaluated.

### Outcome Measures

The primary outcome was the duodenal peristalsis score. Secondary outcomes included total propofol consumption, technical success, hemodynamic stability, complications, recovery times, and endoscopist satisfaction.

### Sample Size

Because data regarding the effects of IV lidocaine on duodenal peristalsis during ERCP were limited, the sample size estimation was based primarily on anticipated differences in the requirement for rescue antispasmodic agents, which were considered a clinically relevant procedural parameter. Previous endoscopic peristalsis studies evaluating topical lidocaine,^11^ together with pilot clinical observations, were used to estimate the expected effect size. Assuming a 2-sided *α* value of 0.05 and 90% power, a total sample size of 120 patients was required.

### Statistical Analysis

Data were analyzed using Statistical Package for the Social Sciences version 22.0 (IBM SPSS Corp.; Armonk, NY, USA). Normality was assessed using the Kolmogorov–Smirnov and Shapiro–Wilk tests. Continuous variables were expressed as mean ± SD or median (interquartile range), as appropriate. Group comparisons were performed using the independent samples *t*-test or Mann–Whitney *U*-test. Categorical variables were analyzed using the chi-square test or the Fisher’s exact test. Repeated hemodynamic measurements were analyzed using repeated-measures analysis of variance or the Friedman test, as appropriate.

Before multivariable logistic regression analysis, univariate analyses were performed to evaluate associations between baseline variables and clinically adequate inhibition of duodenal peristalsis. Variables considered clinically relevant to procedural difficulty, including native papilla status, and variables showing a potential association in univariate analyses (*P* < .10) were entered into the multivariable logistic regression model. Model calibration was assessed using the Hosmer–Lemeshow goodness-of-fit test, and explanatory power was evaluated using Cox and Snell and Nagelkerke *R*^2^ values.

## Results

**Figure 1.**
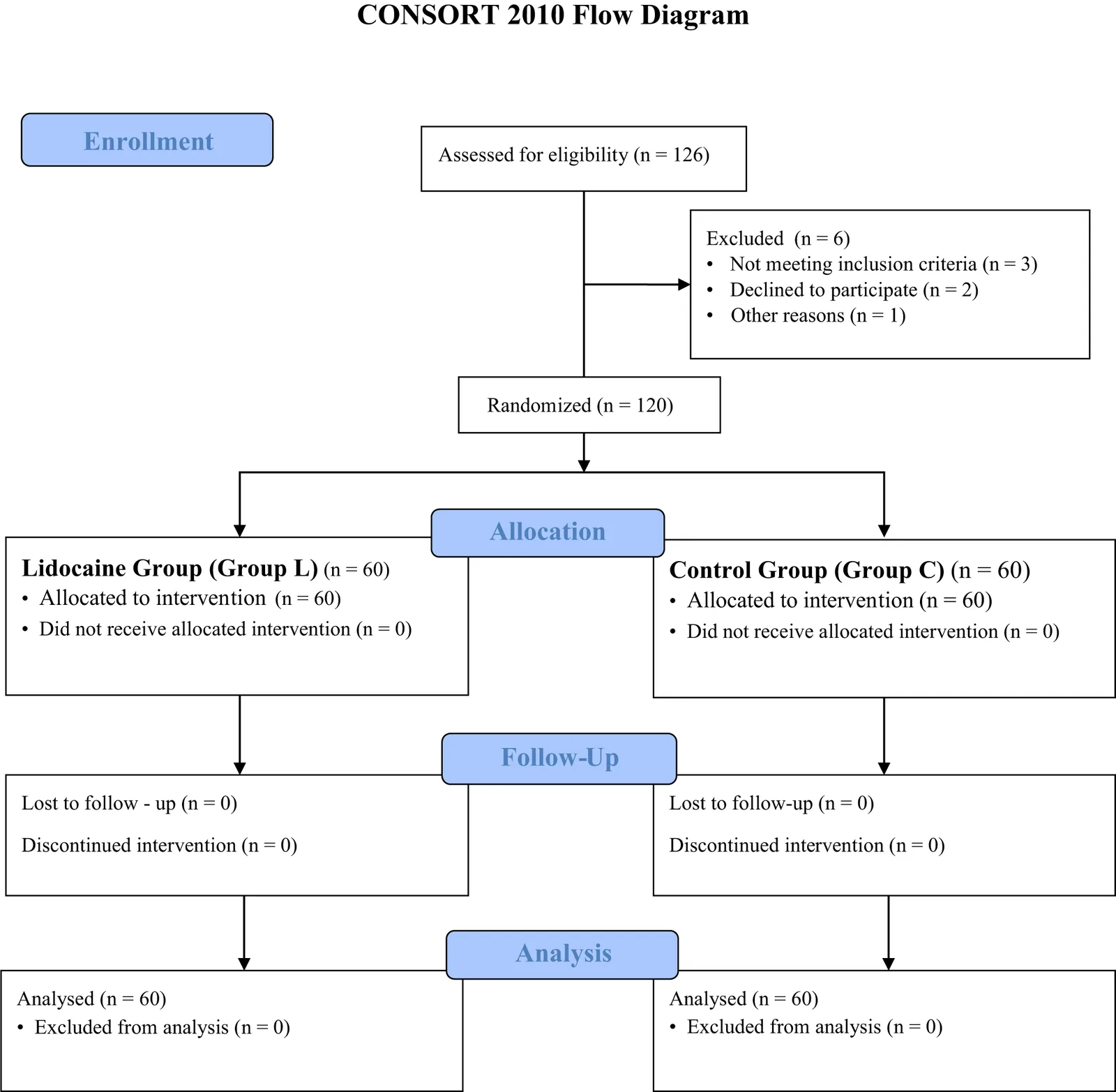
Consolidated Standards of Reporting Trials Flow Diagram of the Study. The diagram illustrates the flow of participants through each stage of the randomized trial, including enrollment, randomization, follow-up, and analysis.

A total of 126 patients were assessed for eligibility. After exclusion of 6 patients who did not meet the inclusion criteria or declined to participate, 120 patients were randomized to the lidocaine (n = 60) and control (n = 60) groups. The flowchart of the study is shown in Figure 1. Demographic and clinical characteristics of the patients were comparable between the 2 groups (Table 1). No significant differences were observed in age, sex, body mass index (BMI), BMI classification, ASA physical status, comorbidities, smoking status, alcohol use, or ERCP indication (all *P* > .05). However, the proportion of patients with a native papilla was significantly higher in the control group than in the lidocaine group (33.3% vs. 16.7%; *P* = .035). In addition, the incidence of sore throat at 24 hours after the procedure was significantly lower in the lidocaine group than in the control group (21.7% vs. 63.3%; *P* < .001) (Table 1).

Procedural outcomes, sedation parameters, and postprocedural complications are presented in Table 2. The procedural duration was significantly longer in the lidocaine group than in the control group (38.5 [IQR: 35.0-41.8] min vs. 35 [IQR: 30-41] min; *P* = .009). Recovery and discharge times were significantly shorter in the lidocaine group (18.5 [16-20] min vs. 27 [25-29] min and 35 [32-38] min vs. 44 [39-48] min, respectively; *P* < .001 for both). Total propofol consumption was significantly lower in the lidocaine group (183 [171.2-192.8] mg vs. 231.5 [219.0-248.2] mg; *P* < .001). Furthermore, the requirement for rescue antispasmodics and the use of hyoscine-N-butylbromide were significantly lower in the lidocaine group (*P* = .018 and *P* = .004, respectively), whereas rescue glucagon use did not differ between the groups (*P* = .99). No significant differences were observed between the groups in cannulation time, primary cannulation success rate, or complication rates such as hiccups, gagging, coughing, body movements, hypotension, bradycardia, hypoxemia, nausea, and vomiting (all *P* > .05). Clinically adequate inhibition of duodenal peristalsis was achieved more frequently in the lidocaine group than in the control group (90.0% vs. 75.0%; *P* = .031). However, the overall ordinal duodenal peristalsis scores did not differ significantly between groups (*P* = .791) (Table 2).

Baseline demographics and clinical characteristics according to duodenal peristalsis inhibition status are presented in Table 3. No significant differences were observed in baseline clinical properties, indications, or demographic parameters between patients with and without clinically adequate duodenal peristalsis inhibition (all *P* > .05) (Table 3).

To evaluate the potential impact of the baseline imbalance in native papilla distribution, a post hoc subgroup analysis was performed among patients with a native papillae (N = 30; control group, n = 20; lidocaine group, n = 10). Among the procedural, sedation-related, and peristalsis outcomes assessed in this subgroup, first-pass success, endoscopist satisfaction, procedure duration, cannulation time, rescue hyoscine-N-butylbromide use, rescue glucagon use, duodenal peristalsis score, and clinically adequate duodenal peristalsis inhibition rates were not significantly different between the groups (all *P* > .05). However, even among patients with native papillae, recovery time (20 [16.0-21.2] minutes vs. 26.5 [25.0-30.0] minutes; *P* < .001), discharge time (36 [33.8-39.0] minutes vs. 46.5 [41.0-48.8] minutes; *P* < .001), and total propofol dose (175.5 [164.5-187.0] mg vs. 241.5 [219.8–255.8] mg; *P* < .001) were significantly lower in the lidocaine group than in the control group (Table 4).

In the multivariable logistic regression analysis evaluating independent predictors of successful peristalsis control, lidocaine use remained independently associated with a higher likelihood of achieving clinically adequate inhibition of duodenal peristalsis (OR, 3.01; 95% CI, 1.02-8.89; *P* = .046). Native papilla status, which was included in the multivariable regression model, was not independently associated with this outcome (OR, 0.46, 95% CI, 0.15-1.37; *P* = .161). No other clinical variables demonstrated a significant independent association (Table 5). The final regression model demonstrated good calibration (Hosmer–Lemeshow test *P* = .602) with a Nagelkerke *R*^2^ value of 0.15 (Table 5).

## Discussion

In the current study, the effects of IV lidocaine on sedative requirements, procedural outcomes, and duodenal peristalsis during ERCP were comprehensively evaluated. The findings suggest that IV lidocaine significantly reduced propofol consumption while maintaining adequate sedation and was associated with greater inhibition of duodenal peristalsis. In addition, the reduction in recovery and discharge times represents a clinically relevant advantage. No significant differences were observed between the groups in hemodynamic parameters. Overall, these findings suggest that IV lidocaine may provide supportive anesthetic-sparing adjunct and peristalsis-modulating effects during ERCP.

Control of duodenal peristalsis during ERCP is essential for both procedural success and safety. Agents commonly used for this purpose, such as hyoscine-N-butylbromide and glucagon, are associated with significant adverse effects.^12^^-^^15^ The reduced need for rescue antispasmodic agents observed in the lidocaine group in the current study suggests a potential procedural benefit. This reduction may help minimize drug-related adverse effects, particularly in patients with cardiac comorbidities and may indirectly contribute to hemodynamic stability. In this respect, the peristalsis control provided by lidocaine may represent an adjunctive procedural effect, even if it does not directly improve technical success. Although a higher rate of clinically adequate inhibition of duodenal peristalsis was observed in the lidocaine group after dichotomization of the peristalsis scores, the original ordinal scores did not significantly differ between the groups. Therefore, these findings should be interpreted cautiously, and the observed physiological effect may be considered supportive rather than definitive evidence of procedural benefit.

One of the most notable findings of the current study was that lidocaine use was associated with significantly higher rates of clinically adequate duodenal peristalsis inhibition, and this association remained significant in the multivariable analysis. Although the exact mechanisms underlying effects of lidocaine on gastrointestinal peristalsis are not fully understood, lidocaine has been proposed to suppress reflex activity through visceral afferent pathways, thereby reducing gastrointestinal peristalsis.^10^^,^^16^ The effects of topical lidocaine on gastrointestinal peristalsis have been previously reported. Suzuki et al^11^ reported significant inhibition of duodenal peristalsis, and Nemoto et al^17^ similarly reported that topical lidocaine application during colonoscopy significantly reduced colorectal spasms. The effects of IV lidocaine on the gastrointestinal system have mainly been investigated in major abdominal surgery, where prolonged infusions have been associated with faster recovery of bowel function, reduced opioid requirements, and improved analgesia.^18^^,^^19^ However, these findings have not been consistent across all clinical settings, as some randomized trials have reported limited benefits.^20^

In this context, it is noteworthy that even a short-term IV lidocaine infusion was associated with a higher rate of clinically adequate duodenal peristalsis inhibition during ERCP, where control of acute duodenal spasm is critical. This effect, together with reduced propofol requirements and decreased use of rescue antispasmodics, suggests that the acute neuromodulatory effects of lidocaine may contribute to procedural peristalsis control. These findings support the hypothesis that the effects of lidocaine may be mediated through short-term neurophysiological modulation during the procedure rather than through long-term effects on bowel function.

Although lidocaine significantly suppressed duodenal peristalsis, technical outcomes such as cannulation time, primary cannulation success rates, and overall endoscopist satisfaction scores were statistically similar between the groups. These findings suggest that although IV lidocaine successfully achieves a quieter duodenal field, this physiological effect may not translate into a measurable mechanical superiority or altered operator perception. Clinically, ERCP performance and endoscopist satisfaction are influenced by a multifactorial interplay that extends far beyond duodenal peristalsis alone, including variant papillary anatomy, baseline procedural complexity, and individual operator experience. This multifactorial nature is also reflected in the significantly longer procedural duration observed in the lidocaine group. Procedural duration during ERCP is heavily influenced by underlying biliary pathology and therapeutic interventions rather than peristalsis control alone. In the current study, the numerically higher proportion of periampullary carcinoma cases in the lidocaine group (18.3% vs. 8.3%) inherently contributed to longer and more technically demanding procedures. Furthermore, the baseline imbalance in the proportion of patients with a native papillae in the control group (33.3% vs. 16.7%) must be considered, as native papilla status is associated with increased cannulation difficulty. Consequently, both procedural duration and endoscopist satisfaction should be interpreted cautiously as outcomes modulated by these baseline clinical complexities, rather than as a direct surrogate of duodenal peristalsis control or procedural conditions alone.

The sedative-sparing effect of IV lidocaine represents another key finding of the current study. This effect is consistent with its known analgesic, antihyperalgesic, and central nervous system–modulating properties.^21^^-^^24^ Previous studies have demonstrated that lidocaine reduces anesthetic and opioid requirements.^25^ In the current study, total propofol consumption was reduced by approximately 21%, whereas BIS values remained within the target range in both groups, indicating preserved sedation quality.^26^ These findings highlight the clinical relevance of anesthetic-sparing effect of lidocaine.

The reduction in propofol dosage, together with shorter recovery times and fewer postprocedural symptoms, suggests that lidocaine may contribute to improved perioperative stability.^27^^-^^30^ In addition, shorter discharge times may enhance clinical workflow, particularly in outpatient settings.^31^ Hemodynamically, no overall significant differences were observed between the groups, and the transient difference in MAP at 10 minutes was considered clinically negligible. These findings support the safety of IV lidocaine in this setting.

Although complication rates were not significantly different between the groups, the relatively low number of adverse events may have limited the ability to detect statistically significant differences. Numerically lower rates of hypoxemia were observed in the lidocaine group, although this finding did not reach statistical significance. In addition, reduced propofol consumption was accompanied by shorter recovery and discharge times, consistent with previous studies reporting anesthetic-sparing effects of IV lidocaine during endoscopic procedures. Therefore, IV lidocaine may provide supportive periprocedural benefits; however, these findings should be interpreted with caution.

Another notable finding regarding postprocedural recovery was the lower incidence of sore throat at 24 hours in the lidocaine group, suggesting potential benefits of IV lidocaine on overall patient comfort. This observation is consistent with recent literature describing the airway-protective and analgesic characteristics of systemic lidocaine. For instance, Jiang et al^32^ reported that a continuous IV infusion of lidocaine reduced the incidence and severity of postoperative sore throat and cough up to 24 hours, potentially through suppression of local mucosal irritation and airway reflex reactivity. During ERCP, the mechanical passage and manipulation of the endoscope inevitably induce pharyngeal stress. In the current cohort, the systemic action of IV lidocaine may have blunted upper airway reflexes, leading to a smoother procedural course with fewer episodes of gagging or coughing. By potentially minimizing this mechanical friction and providing systemic analgesia, IV lidocaine appears to have contributed to reduced pharyngeal soreness, representing a clinically favorable addition to postprocedural patient satisfaction.

The potential risk of local anesthetic systemic toxicity should always be considered when IV lidocaine is administered. In the current study, lidocaine dosing remained within recommended safety limits, patients with conditions associated with increased toxicity risk were excluded, and continuous ECG, blood pressure, oxygen saturation, and BIS monitoring were performed throughout the procedure.^33^ Although no serious adverse events suggestive of lidocaine toxicity were observed, careful patient selection and close periprocedural monitoring remain essential for safe clinical use.

The current study had several limitations. The single-center design and relatively sample size may limit the generalizability of the findings. In addition, different dosing regimens were not compared, long-term outcomes were not assessed, and potential variability in sedation management and operator dependency should be acknowledged. Future multicenter studies with larger sample sizes and varying dosing protocols are warranted to further define the optimal use of lidocaine during ERCP. Moreover, although a subgroup analysis was performed in patients with naïve papillae (N = 30) to address specific procedural complexities, this subgroup was limited by a small sample size. Another important limitation relates to the assessment of duodenal peristalsis. Although the 5-point semiquantitative scoring system used in the current study was adapted from previously published endoscopic studies, it has not been formally validated and interobserver reliability has not been established. Because duodenal peristalsis was assessed by a single blinded endoscopist, measurement subjectivity cannot be completely excluded. Furthermore, the dichotomization of the ordinal peristalsis score may have reduced data granularity and influenced the robustness of the statistical analysis. Therefore, findings related to duodenal peristalsis and clinically adequate inhibition of duodenal peristalsis should be interpreted with caution, and future studies should validate standardized scoring systems and assess interobserver agreement.

## Conclusion

The current study suggests that IV lidocaine may serve as a supportive anesthetic-sparing adjunct during ERCP performed under deep sedation. Lidocaine administration was associated with reduced propofol requirements, higher rates of clinically adequate inhibition of duodenal peristalsis, and shorter recovery and discharge times. However, major procedural outcomes, including cannulation time, technical success, and endoscopist satisfaction, were comparable between groups.

In addition, no major differences in hemodynamic parameters or adverse events were observed between the groups, supporting the feasibility of IV lidocaine use when administered with appropriate patient selection and monitoring. Nevertheless, given the limitations related to the nonvalidated peristalsis scoring system and the dichotomized analysis of clinically adequate inhibition of duodenal peristalsis, these findings should be interpreted with caution. Further large-scale, multicenter randomized controlled trials with larger sample sizes are warranted to better define the clinical implications and optimal role of IV lidocaine during ERCP. Specifically, future highly powered clinical trials focusing on patients with native papillae are strongly recommended to definitively isolate the structural and therapeutic efficacy of systemic lidocaine on initial cannulation dynamics and targeted peristalsis control.

## Figures and Tables

**Table 1.** Baseline Demographic and Clinical Characteristics of the Control and Lidocaine Groups

	Control (n = 60)	Lidocaine (n = 60)	*P*
Age (years)	58.8 ± 12.3	60.2 ± 12.5	.522
Gender			
Male	23 (38.3)	26 (43.3)	.577
Female	37 (61.7)	34 (56.7)	
BMI (kg/m^2^)	28.3 ± 4.6	28.6 ± 4.8	.695
BMI classification			
Normal (<25.0)	16 (26.7)	15 (25.0)	.853
Overweight (25.0-29.9)	22 (36.7)	20 (33.3)	
Obese (≥30.0)	22 (36.7)	25 (41.7)	
ASA classifications			
I	15 (25.0)	10 (16.7)	.516
II	29 (48.3)	31 (51.6)	
III	16 (26.7)	19 (31.7)	
Comorbidities			
HT	8 (13.3)	10 (16.7)	.609
DM	8 (13.3)	6 (10.0)	.570
COPD	3 (5.0)	3 (5.0)	.99
CAD	10 (16.7)	12 (20.0)	.637
Smoking	15 (25.0)	8 (13.3)	.104
Alcohol	10 (16.7)	8 (13.3)	.609
ERCP indications			
Choledocholithiasis	35 (58.3)	37 (61.7)	.116
Malignant or benign biliary strictures	20 (33.3)	12 (20.0)	
Periampullary carcinoma	5 (8.3)	11 (18.3)	
Native papilla	20 (33.3)	10 (16.7)	.035*
First pass success	34 (56.7)	29 (48.3)	.361
Sore throat (24 hours)	38 (63.3)	13 (21.7)	<.001*
Endoscopist satisfaction	8.6 ± 0.5	8.4 ± 0.4	.146

ASA, American Society of Anesthesiologists; BMI, body mass index; CAD, coronary artery disease; COPD, chronic obstructive pulmonary disease; DM, diabetes mellitus; ERCP, endoscopic retrograde cholangiopancreatography; HT, hypertension.

**Table 2.** Comparison of Procedural Outcomes, Sedation Characteristics, and Complications between Control and Lidocaine Groups

	Control (n = 60)	Lidocaine (n = 60)	*P*
Procedure duration (min)	35 (30-41)	38.5 (35.0-41.8)	.009*
Recovery time (min)	27 (25-29)	18.5 (16-20)	<.001*
Discharge time (min)	44 (39.0-48.0)	35 (32-38)	<.001*
Cannulation time (sec)	120 (60.0-157.5)	94.5 (60.0-157.5)	.751
Total propofol (mg)	231.5 (219.0-248.2)	183 (171.2-192.8)	<.001*
Rescue hyoscine-n-butylbromide	14 (23.3)	3 (5.0)	.004*
Rescue glucagon	3 (5.0)	3 (5.0)	.99
Complications			
Hiccup	2 (3.3)	1 (1.7)	.99
Gagging/coughing	4 (6.7)	2 (3.3)	.679
Body movements	3 (5.0)	3 (5.0)	.99
Hypotension	7 (11.7)	5 (8.3)	.543
Bradycardia	5 (8.3)	3 (5.0)	.717
Hypoxemia	9 (15.0)	3 (5.0)	.125
Nausea and vomiting	5 (8.3)	3 (5.0)	.717
Duodenal peristalsis score	4 (3.2-5.0)	4 (4-5)	.791
Clinically adequate duodenal peristalsis inhibition	45 (75.0)	54 (90.0)	.031*

IQR, interquartile range.*Statistically significant P values (P < .05) are shown in bold.

**Table 3.** Comparison of Baseline Characteristics According to Duodenal Peristalsis Inhibition Status

	Inhibition (−) (n = 21)	Inhibition (+) (n = 99)	*P*
Age (years)	56.4 ± 14.2	60.2 ± 11.9	.205
Gender			
Male	10 (47.6)	39 (39.4)	.486
Female	11 (52.4)	60 (60.6)	
BMI (kg/m^2^)	29.1 ± 4.0	28.3 ± 4.8	.540
BMI classification			
Normal (<25.0)	3 (14.3)	28 (28.3)	.289
Overweight (25.0-29.9)	10 (47.6)	32 (32.3)	
Obese (≥30.0)	8 (38.1)	39 (39.4)	
ASA classifications			
I	3 (14.3)	22 (22.2)	.675
II	12 (57.1)	48 (48.5)	
III	6 (28.6)	29 (29.3)	
Comorbidities			
HT	5 (23.8)	13 (13.1)	.309
DM	3 (14.3)	1 (11.1)	.710
COPD	1 (4.8)	5 (5.1)	.000
CAD	2 (9.5)	20 (20.2)	.358
Smoking	4 (19.0)	19 (19.2)	.99
Alcohol	3 (14.3)	15 (15.2)	.99
ERCP indications			
Choledocholithiasis	13 (61.9)	59 (59.6)	.097
Malignant biliary obstruction	8 (38.1)	24 (24.2)	
Periampullary carcinoma	0	16 (16.2)	
Native papila	8 (38.1)	22 (22.2)	.127
First pass success	12 (57.1)	51 (51.5)	.639
Sore throat (24 hours)	11 (52.4)	40 (40.4)	.313

Continuous variables are presented as mean ± SD, whereas categorical variables are expressed as number (percentage). Statistical significance is defined as *P* < .05.

ASA, American Society of Anesthesiologists; BMI, body mass index; CAD, coronary artery disease; COPD, chronic obstructive pulmonary disease; DM, diabetes mellitus; ERCP, endoscopic retrograde cholangiopancreatography; HT, hypertension.

**Table 4.** Subgroup Analysis of Procedural and Peristalsis Outcomes in Patients with Native Papillae

	Control (n = 20)	Lidocaine (n = 10)	*P*
First-pass success	9 (45.0)	4 (40.0)	.999
Endoscopist satisfaction	14 (70.0)	6 (60.0)	.690
Procedure duration (minutes)	37.5 (31.0-41.5)	39 (34.2-41.2)	.422
Recovery time (minutes)	26.5 (25-30)	20 (16.0-21.2)	<.001*
Discharge time (minutes)	46.5 (41.0-48.8)	36 (33.8-39.0)	<.001*
Cannulation time (seconds)	160 (108.8-200.0)	200 (170-280)	.109
Total propofol (mg)	241.5 (219.8-255.8)	175.5 (164.5-187.0)	<.001*
Rescue hyoscine n-butylbromide	5 (25.0)	0	.140
Rescue glucagon	1 (5.0)	0	.999
Duodenal peristalsis score	4 (2-5)	4 (4-4)	.746
Clinically adequate duodenal peristalsis inhibition	13 (65.0)	9 (90.0)	.210

Continuous variables are presented as median (25th-75th percentiles), whereas categorical variables are expressed as number (percentage). Statistical significance is defined as *P* < .05.

IQR, interquartile range.

**Table 5.** Multivariable Logistic Regression Analysis of Factors Associated with Duodenal Peristalsis Inhibition

	OR	95% CI	*P*
Age (years)	1.03	0.99-1.07	.144
Sex (female)	1.62	0.58-4.50	.353
BMI (kg/m^2^)	0.96	0.86-1.07	.418
ASA classificiation			
I	Reference		
II	0.34	0.08-1.51	.155
III	0.54	0.11-2.67	.451
Smoking	1.18	0.33-4.28	.796
Native papila	0.46	0.15-1.37	.161
Study group			
Control	Reference		
Lidocaine	3.01	1.02-8.89	.046*

Hosmer–Lemeshow test, *χ*^2^ = 6.40; *P* = .602; Cox and Snell *R*^2^, 0.09; Nagelkerke *R*^2^, 0.15.

ASA, American Society of Anesthesiologists; BMI, body mass index; OR, odds ratio; *R*^2^, coefficient of determination.

*Statistically significant P values (P < .05) are shown in bold.

## Data Availability

The data that support the findings of this study are available on request from the corresponding author.
